# A developmental angle to understanding the mechanisms of biased cognitions in social anxiety

**DOI:** 10.3389/fnhum.2013.00846

**Published:** 2014-02-25

**Authors:** Simone P. W. Haller, Kathrin Cohen Kadosh, Jennifer Y. F. Lau

**Affiliations:** ^1^Department of Experimental Psychology, University of OxfordOxford, UK; ^2^Department of Psychology, Institute of Psychiatry, King's College LondonLondon, UK

**Keywords:** social anxiety, cognitive bias, attention, interpretation, neurocognitive development, fMRI, children and adolescents

## Introduction

Social anxiety disorder (SAD) is debilitating and common, affecting 7.3–12.1% of the population (e.g., Wittchen et al., 1999; Kessler et al., 2005). Age-of-onset data show that SAD symptoms are often first experienced in late childhood or adolescence (Kessler et al., 2005). While adolescence is a period when many typical social fears and worries emerge, major questions remain as to why some youths are more vulnerable to experiencing persistent and impairing social anxiety. A key gap in current theoretical models of SAD etiology is an understanding of the mechanisms by which risk factors are expressed during development. In this opinion paper, we address this gap by first discussing the nature of age-typical increases in social fears and worries in the transition to adolescence and outlining possible brain-based developmental mechanisms by which these arise. Next, we discuss how these age-typical changes in neurocognitive functioning might, in a subset of adolescents, enable maladaptive processing biases in relation to social cues to emerge or be exacerbated. These processing biases may, in turn, contribute to the onset of persistent social anxiety.

## Adolescence: a period of age-typical increases in social fears and worries

Adolescence is a transitional period demarcated by the onset of puberty, and ending with the assumption of a stable adult role (Lerner and Steinberg, 2004). This transitional period involves substantial physiological and psychological changes, currently understood to be orchestrated by a combination of experience-dependent and biologically programmed regulation of gene expression (Nelson et al., 2005; Gajados et al., 2010). Central to adolescent developments are hormonal changes associated with puberty. These likely initiate a cascade of morphological and neural maturations, which significantly impact on cognition and information processing (Sisk and Foster, 2004; Blakemore et al., 2010; Goddings et al., 2013). Of particular interest in the context of this opinion paper are the effects of these maturational changes on the processing of affective and social stimuli. In the last decade, there has been a surge in investigations of typical changes in the functioning of limbic and prefrontal networks across development, especially during social-affective processing (e.g., Pfeifer and Blakemore, 2012). Cross-sectional comparisons of functional neuroimaging data from multiple child/adolescent/adult age groups may lend insight into how typical developmental changes in the brain can give rise to adolescent-typical behaviors of heightened “emotionality” and “sociality”. In turn, these may explain age-associated changes in social fears and worries in adolescence.

What typical neurodevelopmental changes might increase “emotionality” (that is, increased avoidance of threats and approach of rewards) across age? There is now a convincing corpus of data available documenting changes in the sensitivity of subcortical regions involved in basic processing of threat and reward such as the amygdalae and striatum. These data broadly suggest a peak in the neural response to monetary rewards, emotional faces and peer feedback in early to mid-adolescence, before decreasing toward adulthood (e.g., Monk et al., 2003; Ernst et al., 2005; Galvan et al., 2006; Hare et al., 2008; Van Leijenhorst et al., 2010; Pfeifer et al., 2011; Somerville et al., 2011; Chein et al., 2012; Gee et al., 2013). More protracted changes have also been noted in regulatory regions involved in modulating arousal. The few cross-sectional functional magnetic resonance imaging (fMRI) studies of adolescents have consistently found differences in medial and lateral functional subdivisions of the prefrontal cortex (PFC) in response to emotionally provocative stimuli between adolescents and adults (e.g., Yurgelun-Todd and Killgore, 2006; Masten et al., 2009; Gunther Moor et al., 2010, 2012; Lau et al., 2011; Pitskel et al., 2011; McRae et al., 2012). However, the directionality of these developmental differences is not always consistent, possibly because of variations associated with the social-motivational context of the task (see Crone and Dahl, 2012 for in-depth discussion). Nonetheless, additional recruitment of medial and lateral PFC regions in older age groups (relative to younger participants) has been tentatively interpreted as reflecting an increased ability to recruit these regions to effectively down-regulate subcortical arousal with age (e.g., Gunther Moor et al., 2010; Casey et al., 2011). Further support for this interpretation comes from studies showing that regulatory functional connectivity between PFC and subcortical regions continues to mature throughout adolescence (Hare et al., 2008; Pitskel et al., 2011; Gee et al., 2013). Synthesis of these data suggests that protracted maturation of prefrontal engagement together with a heightened reactivity of limbic regions to threatening and rewarding stimuli may be responsible for increased emotional responses in adolescence. Notably, these studies of age-associated functional differences occur against a backdrop of structural developments in these regions too, with data pointing to localized linear and non-linear restructuring as well as further integration within networks (e.g., Giedd et al., 1999; Paus et al., 1999; Sowell et al., 1999; Gogtay et al., 2004; Dennis et al., 2013).

What typical neurodevelopmental changes might increase “sociality” (that is, increased motivational salience of peers and understanding of complex social situations) across age? Continuous development throughout adolescence has been documented in the network involved in the understanding of others' behavior in terms of motivations, thoughts and feeling states (“mentalizing”) (Blakemore, 2008; Mills et al., 2014). Developmental studies of mentalizing have consistently found a (relative) decrease in anterior/dorsal medial PFC activity and increase in posterior-temporal areas (such as the temporo-parietal junction and superior temporal sulcus) in response to tasks requiring mental state attribution when comparing early/mid adolescent to adult groups (Wang et al., 2006; Blakemore et al., 2007; Burnett et al., 2009; Guroglu et al., 2011). More recent studies have employed multiple adolescent age groups and have confirmed a continuous shift in functional contributions from frontal to temporal areas across adolescence whilst engaged in thinking about mental states (Gunther Moor et al., 2011; Van den Bos et al., 2011)—findings that have been suggested to reflect increased automaticity of engaging in mentalizing across adolescence (e.g., Blakemore, 2008; Van den Bos et al., 2011). Presumably such neurocognitive changes prepare the adolescent for navigation in a novel and possibly more complex social world.

In summary, changes in brain networks engaged by social-affective stimuli across adolescence may result in greater affective responding and a greater engagement with, and understanding of, complex interpersonal situations. These age-typical changes may, on the one hand, allow for more flexible responses enabling the adolescent to adapt rapidly to changing social contextual demands (Crone and Dahl, 2012). Yet, on the other hand, these normative brain developments, which change the perception of and importance placed on the social world, may increase social fears and worries. This may be particularly crucial given that some social environments are changing. For example, school transitions often mean longer time in school and greater workload, as well as more time spent interacting with peers socially and academically. Such changes may increase the opportunities for new academic pressures to emerge, and new peer groups and hierarchies to be formed.

## Adolescence: a period of precipitating individual differences in social anxiety

While we suggest that most adolescents will experience age-associated increases in social fears and worries, in a minority of adolescents, more distressing forms of social anxiety may also emerge and persist. A key question is what makes these individuals different? Similar to adult models of SAD, theoretical considerations of child and adolescent SAD have emphasized biases in information processing (Clark and Wells, 1995; Rapee and Heimberg, 1997; Ollendick and Hirshfeld-Becker, 2002; Jarcho et al., 2013). *Attention* biases, that is, systematic differences in orienting to threat cues, have been documented in socially anxious children and adolescents (Stirling et al., 2006; Roy et al., 2008) and even in at-risk infants (offspring of socially anxious mothers) as young as 10 weeks (Creswell et al., 2008). Biases in the *interpretation* of ambiguous social information have also been found in socially anxious youths (Haller et al. A novel picture-based tool for measuring interpretation biases in adolescents, manuscript in preparation; Miers et al., 2008) although, interestingly, linkages between biased interpretations and symptoms are less consistently found in younger children (Waters et al., 2008; In-Albon et al., 2009; Creswell et al., 2013). It may either be that current measurement tools are not suitable for detecting interpretation biases in younger populations or that interpretation biases do not mature as risk factors until later in adolescence. Finally, biases in *expectations* of the outcomes of social-evaluative situations also characterize socially anxious individuals and at-risk populations (Cartwright-Hatton et al., 2003, 2005; Pass et al., 2012).

Recent fMRI studies have suggested that in adults, SAD-linked cognitive biases may be associated with individual differences in brain activity. Thus, biases in attention and the tendency to perceive ambiguous social cues such as neutral facial expressions as negative have been linked to impaired regulatory recruitment of fronto-amygdalae circuits and increased emotion-related neural responses of limbic areas in SAD individuals (e.g., Cooney et al., 2006; Blair et al., 2008). The few studies investigating the neural substrates of information processing and cognitions in adolescents with social concerns mostly find similar results (Killgore and Jurgelun-Todd, 2005; Pérez-Edgar et al., 2007; Guyer et al., 2008, 2014).

How might age-normative neural changes in social-affective regions impact on the expression of individual differences in cognitive biases thereby increasing vulnerability to SAD in adolescence? We suggest that age-typical changes in emotionality and sociality in adolescence may magnify differences across individuals such that those who already fall at the end of the continuous distribution shift further toward the extreme end. Speculatively, this can occur through two routes. First, developmental changes in the sensitivity of the “emotional brain” may further amplify attention and expectancy biases for potential threat cues. Bi-directional interactions between pre-existing cognitive biases and the plasticity of the adolescent brain may serve to amplify negative effects over time. Pre-existing cognitive biases will affect functional restructuring by biasing incoming information to further sensitize socio-affective networks. Hence, individuals with a pre-existing tendency to attend to negative aspects of social cues or situations—or to expect negative outcomes from these—may experience these to a greater degree and, to alleviate distress, may engage in maladaptive behavioral strategies such as avoidance. This will set up a vicious cycle, which, over time, reinforces these pre-existing maladaptive biases.

Secondly, developmental changes in the “social brain” may act as a vehicle for the expression of biases at the level of interpretation. As increased mentalizing abilities (being able to generate more “mental explanations” for others' behavior) emerge. The emergence of increased mentalizing abilities across adolescence may result in an increase in perceived complexity and ambiguity of daily social situations. Specifically, as these maturational brain developments are paralleled by increases in time spent with peers, this change in perception of social interactions may “bring out” in some individuals the tendency to interpret socially ambiguous cues in a more negative manner. This could also explain findings of why interpretation biases are not consistently found in younger populations—it may be that such biases in interpretation only become evident once these socio-cognitive capacities are attained.

In order to empirically investigate these hypotheses, studies need to assess whether certain neurocognitive factors characterize individuals with social anxiety at particular ages or at particular pubertal developmental stages. This can be done by looking at SAD-linked processing biases in individuals with high and low social anxiety (or with and without SAD) across different developmental age groups. Our prediction is that while attention, expectancy and interpretation biases at the behavioral and neural level characterize all participants with high levels of social anxiety (or who meet criteria for SAD), these group differences will prove to be far stronger in adolescents than in children. Moreover, we would predict that these age-by-group interactions are mediated by changes in “emotionality” and “sociality”. Such hypotheses await future empirical investigations.

## Conclusion

This opinion paper has highlighted how neuro-scientific insights on the level of normative functional changes during adolescence can generate novel hypotheses about the mechanisms underlying the emergence or exacerbation of individual differences related to social anxiety. We have described ways in which normative neurodevelopmental progressions could magnify pre-existing cognitive biases in attention, interpretation and expectations that are characteristic of persistent and impairing social fears and worries. We further provided directions as to how these hypotheses should be empirically investigated. Adolescence as a time of increased plasticity may also be an optimal time for administering neurocognitive interventions (e.g., Cohen Kadosh et al., 2013; Lau, 2013) as the exposure to specific adaptive or corrective experiences may result in long-term effects on neural architecture. Understanding the mechanisms by which normative neurodevelopmental changes may drive the expression of risk factors linked to social anxiety can extent current theoretical models of SAD and, in parallel, inform when early interventions should be effectively applied.
